# Two-Layer Fragile Watermarking Method Secured with Chaotic Map for Authentication of Digital Holy Quran

**DOI:** 10.1155/2014/803983

**Published:** 2014-05-29

**Authors:** Mohammed S. Khalil, Fajri Kurniawan, Muhammad Khurram Khan, Yasser M. Alginahi

**Affiliations:** ^1^Center of Excellence Information Assurance, King Saud University, P.O. Box 92144, Riyadh 11653, Saudi Arabia; ^2^IT Research Center for the Holy Quran and Its Sciences (NOOR), Taibah University, Madinah 41477, Saudi Arabia; ^3^Department of Computer Science, College of Computer Science and Engineering, Taibah University, Madinah 41477, Saudi Arabia

## Abstract

This paper presents a novel watermarking method to facilitate the authentication and detection of the image forgery on the Quran images. Two layers of embedding scheme on wavelet and spatial domain are introduced to enhance the sensitivity of fragile watermarking and defend the attacks. Discrete wavelet transforms are applied to decompose the host image into wavelet prior to embedding the watermark in the wavelet domain. The watermarked wavelet coefficient is inverted back to spatial domain then the least significant bits is utilized to hide another watermark. A chaotic map is utilized to blur the watermark to make it secure against the local attack. The proposed method allows high watermark payloads, while preserving good image quality. Experiment results confirm that the proposed methods are fragile and have superior tampering detection even though the tampered area is very small.

## 1. Introduction and Background

Increasing usage and production of handheld devices and smart-phone have pushed Muslim community to create the Holy Quran in the digital form. Thus, authentication of digital Quran becomes an emerging issue because the current digital Quran that is mostly in mobile applications is unverified. Even though the digital Quran is verified by the Islamic authority, still there is a problem such as falsifying some parts of Quran's verses. In such case, the readers are unable to validate the verse, whether it is correct or fake, due to an accidental typo or an intentional act.

History tells that according to investigation in July, 2005, several secret scanned documents of World War II at the National Archives have been altered during or after the 2000s [1]. Hence, protecting a digital document such as the Holy Quran is important nowadays. In this regard, this study proposes a solution to protect the digital Quran and to localize the tamper region on the digital Quran images if present. The proposed solution is inspired by the digital watermarking concept. Digital watermarking means that the digital data embedded with a predefined authentication code remains undetectable to human eye but effortlessly identified by the specified algorithm. The major focus is to shield the integrity, security, and fidelity of the digital content such as that of the digital watermarking promisingly applicable for the present electronically driven world [2]. Particularly, a schema known as fragile watermarking has attracted great attention for authentication and integrity of the digital content [3].

There are several known requirements for a fragile watermarking method that must be considered including detecting common image forgery, geometric transformations, signaling elimination of original image, putting new objects, and notifying once image processing operations present. Moreover, it is better to authenticate the media without referring to the original image; this is known as blind detection [4]. Numerous methods recognized that attacks such as the copy-and-paste [5], vector quantization [6], the Holliman-Memon counterfeiting attack [7], or collage attack [8, 9] should be considered by the fragile watermarking method.

It is known that watermarking methods alter the host image after inserting the authentication code. This situation is unsuitable for sensitive applications such as medical imaging and military purpose [11]. However, most of the image applications can accept the degradation of the image quality as long as the original and watermarked images are perceptually comparable or the content is not influenced. Thus, watermarking the Holy Quran should be done carefully to avoid any influence on the verses.

## 2. Related Works

Digital watermarking is generally categorized into three kinds named fragile, semifragile, and robust watermarking. Fragile watermarking has the characteristic of being easily broken even if small forgery is present. Quite similar to fragile watermarking, semifragile watermarking has small difference, which is being robust to nonhuman forgery such as JPEG compression. Attractiveness of JPEG compression becomes a key of the exploration aspect on semifragile watermarking. The last category named robust watermarking is intended to make it hard to break against any tamper activities. Thus, robust watermarking is suitable for copyright protection of the digital images [12]. Meanwhile, the fragile and semifragile watermarking are commonly applied for authentication of the multimedia content, including video, audio, and still image [13, 14]. Some watermarking methods in the literature show that it can localize the tamper regions, and other methods are only able to inform if the still image is authentic or tampered.

Two concepts, namely, pixel-based and block-based approach, are adopted in watermarking techniques when handling the host image. Pixel-based concept treats the host image pixel by pixel to embed the watermark. The block-based concept treats the host image block by block. These approaches, pixel-based and block-based, can be implemented in two domains known as spatial and frequency domain [11–30]. Watermarking technique in the spatial domain has several security issues other than the frequency domain, including the following.

- Such techniques work on very limited space of an image at the pixel level, depending on the color depth.
- Authentication can be easily passed using some image distortion such as compression or noise.
- Natural statistical property in the color image that has linked components in the color information (e.g., RGB and CMYK) would be distorted after watermarking process makes it open to image attacks.

Watermarking method in pixel-based concept [13, 26–28] is exposed to the brute-force attack because the watermark is commonly hidden into the least significant bits (LSBs) [31]. In addition, such method is suitable only to localize the image forgery on some of the most significant bits. On the other hand, the block-based concept has issues for parameter of the block sizes and watermark payloads. Some experiments are required to determine the proper parameter that facilitates acceptable tamper detection while maintaining the image quality. However, the main drawback in block-based concept is being unable to locate the tampered pixels accurately; this might be important for specific applications such as in the military communication [32]. In addition, Preda [14] found that the latest watermarking methods are susceptible to forging attacks and inaccurate when dealing with unintentional image tampering.

Recent fragile watermarking methods are reviewed to comprehend the state of the art. Many researchers focused on watermarking method for binary image [33] and grayscale image [34–37] and according to [38, 39] only limited are concerned about color images. Even though their method is robust with minimum complexity, the original image must be present during authentication process or extracting the watermark information.

Wong [40] introduced a block-based approach along with the public-key scheme. The host image is presented as LSB-zeroed prior to embedding the binary watermark image. Such method has high security and can handle crop and scale attacks. Their block-based approach does not have any correlation between the neighboring blocks or any blocks within the watermarked image. Thus, this method is exposed to several attacks, including vector quantization, cover-up, and transplantation. Wu and Liu proposed DCT-based watermarking method [41] that embeds a binary watermark into the DCT coefficients. A look-up table is defined and utilized to map the DCT coefficients into zero or one. There are two issues in their method. First, they must transmit the look-up table in a secure channel to perform authentication everywhere. Second, their block-wise approach is independent and hence vulnerable to the same attacks like Wong's method [40]. Li et al. [42] proposed a block-wise method that has dependence between neighboring blocks. Neighboring blocks are combined together to establish dependence among them. The authentication information is extracted from the host image as a binary feature map. Authentication can be done without the original image. They claimed that the method was resistant to cover-up attacks and vector quantization. Unfortunately, the watermark is embedded into LSB that is known weak to brute-force attack. Moreover, contextual dependence that was generated based on deterministic information between blocks is vulnerable to transplantation attack because the contextual dependence is established based on deterministic information. Li et al. [43, 44] tried to avoid transplantation attack and reported that their method provided tamper detection at the pixel level. However, their method worked on spatial domain, which is also less secure to brute-force attack. Barreto et al. [45] focused on transplantation attack; they generated the watermark from a block along with their neighbor using hash function plus some random data. It makes the watermark information nondeterministic and distinctive. Nevertheless, accuracy of the tampering detection is influenced by the block size. In addition, their method is applied on spatial domain and hence vulnerable to brute-force attack. Other researchers attempt to avoid spatial domain by introducing transform-domain schemes [46–48]. Winne at el. [46] watermarked the coefficient of high-frequency subbands of luminance component. Their method achieved better localization and less embedding distortion. Quite similar [48], generating the watermark from LL component and embedding it into LL component too, The watermarked image was reported imperceptible by Xie and Arce [48]. Fridrich et al. [49] considered quantized DCT coefficients to generate the watermark. Then, the watermark is embedded into DCT coefficients. However, their method is not intended to locate the tampering pixels but rather it can tell whether the image is authentic or has been tampered.

As discussed above, the performance of watermarking method can be analyzed through the image quality metrics and performing image attacks. For example, fragility of the watermark can be evaluated using collage attack [4]. In such attack, authenticated blocks of known watermarked image are copied into another image to create forgery content that can pass the authentication process. In the literature, a watermark hidden in wavelet domain shows resistance to brute-force attack [13, 29, 30]. Moreover, imperceptibility of the watermark has reported promisingly with satisfactory PSNR (peak signal-to-noise ratio) in the wavelet-based strategy [12, 15, 16, 25–30]. Hence, wavelet-based strategy has been recognized to shield the digital content against forgery. In this regard, the proposed method prefers wavelet-based strategy to achieve better authentication schema. In addition, chaotic maps [50] have appealed further attention to improve the digital watermarking [51–53]. It is because chaotic maps have properties that are sensitive to initial and parameter values and show chaotic behavior. In this regard, chaotic maps are considered in this study to increase the security of proposed fragile watermarking.

The contributions of this paper are a novel fragile watermarking method that utilized the discrete wavelet transforms (DWT) prior to embedding the watermark and chaotic maps to encrypt the watermark information. This method is applied to protect and authenticate the digital Holy Quran and to be able to locate the tamper region if present.

The rest of the paper is structured as follows: Section 2 explains the proposed watermarking method in the wavelet domain and illustrates the diagrams of embedding and authenticating the method, Section 3 covers the experimental results, and the last section concludes the paper.

## 3. The Proposed Watermarking Method in the Wavelet Domain

**Figure 1:**
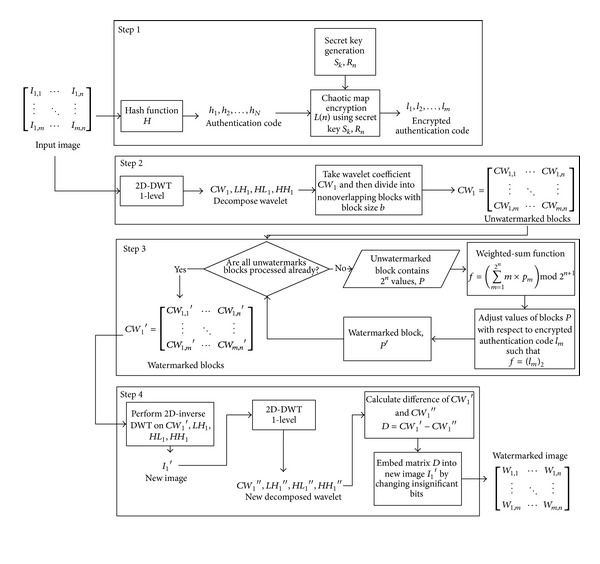
Diagram of the proposed embedding method.

**Figure 2:**
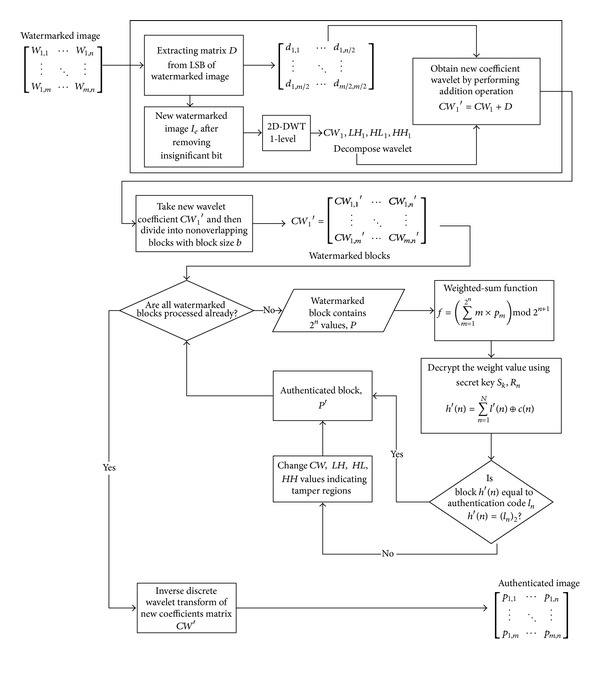
Diagram of the proposed authentication method.

This section describes the proposed fragile watermarking method.  Figure 1 presents the diagram of the proposed embedding process and Figure 2 shows the diagram of the proposed authentication process. The proposed watermarking method is secured by using the chaotic map to blur the authentication code. Hence, intruder is impossible to generate correct authentication code even if they know the initial parameter of the chaotic map because it has random-like behavior.

Chaotic maps are attractive because a small difference in the initial condition would produce a huge difference and they have large variation range [54]. Such characteristics fulfill the classic Shannon's theory for information hiding [55]. Recently, chaotic maps have become popular because they have been proved to enhance the security for information hiding [56]. In this study, two chaotic maps are combined to encrypt the watermark information prior to embedding into the wavelet domain. First chaotic map is used to produce a sequence key and the second is used for data encryption. Such combination offers the subsequent advantages, including being resilient to the fixed length of word that influences by the chaotic sequence, it greatly volatile and it resistant to attacks [54].

The major idea behind this study is to embed the authentication code in the first level of 2D Daubechies discrete wavelet transform. The DWT is a distinguished transformation method that has drawn attention particularly because of the image compression (JPEG2000). 2D DWT used high-pass and low-pass filters to decompose the image into wavelet coefficient, horizontal, vertical, and diagonal details. The DWT-based is considered in this paper rather than DCT- based as the wavelet transform mimics the human vision system (HVS) more similar than the DCT [57]. DWT does not decompose and process the image block by block thus minimizing the image artefacts unlike DCT. DWT clearly splits high- and low-frequency information with respect to pixel by pixel basis [14]. Embedding the watermark into the transform values will only alter the image locally since DWT has a spatial frequency locality characteristic. It is known that changing the coefficients in the wavelet domain is more likely to be undetectable contrasting DCT and FFT. DWT also offers spatial and frequency description of an image. Hence, DWT provides a good basis for hiding the watermark while preserving the image quality [14].

The proposed method is designed to work block by block on the wavelet domain. Each block that consists of the wavelet coefficients is processed consecutively with encrypted watermarks entirely over the image. Hence, particular blocks are able to share duplicate authentication code and create relation between those blocks. This relation makes it hard for the intruder to tamper the watermarked image without breaking the watermarks.

### 3.1. Encryption of the Authentication Code Based on Chaotic Map

Each image of the digital Holy Quran is different for each page. This is because a single page of the Quran image is formed by numerous unique verses. In addition, Quran images include a border to prettify the pages; the border is commonly exclusive among the digital Quran. Hence, such characteristics make the Quran image have a great chance to produce the secret key that is required by the chaotic map. The chaotic trajectory is sensitive to its parameter value and the initial condition. In this regard, random pixel values are selected from the Quran image. The encryption of authentication code begins by secret key's generation, then followed by the encryption process. The steps are detailed as follows.

(i) Take randomly one pixel value from *x*-axis and one pixel value from *y*-axis of the Quran image, defined as *p*_*x*_ and *p*_*y*_. Nonzero pixels are considered for those values.
(ii) Apply equation below to obtain *Q* value that is later used for generating the parameter value and initial condition, the equation being as follows:
  (1) $$\begin{array}{c} Q=\frac{\left|p_{x}-p_{y}\right|}{N},\;0\leq Q\leq 1, \end{array}$$
  where *N* is the pixels amount of the Quran image.
(iii) Equations below proposed by Phan [21] are utilized to generate the parameter value:
  (2) $$\begin{array}{c} S_{k}=\{\begin{array}{cc} 3+Q, & Q>0.57,\\ 3.75+\left(0.43-Q\right), & Q\leq 0.57. \end{array} \end{array}$$
(iv) Initial condition for the chaotic map is defined using equation as follows:
  (3) $$\begin{array}{c} R_{n}=\left|\left\lceil Q\right\rceil -Q\right|,\;0<R_{n}\leq 1. \end{array}$$
(v) Generate a sequence of real numbers using logistic map. That sequence is later used for parameter value of Henon map,
  (4) $$\begin{array}{c} R_{n+1}=\mu R_{n}\left(1-R_{n}\right),\;n=1,2,3,\ldots , \end{array}$$
  where *n* is the map iteration index and the previously calculated secret keys *S*_*k*_ and *R*_*n*_ are used as the parameter value and initial condition of the logistic map, respectively. The logistic map has proven to be very sensitive to initial value, nonconvergent, and nonperiodic when 3.57 < *μ* ≤ 4.0 [58].
(vi) Henon map [18, 22] is employed to encrypt the authentication code; the generalized equation is presented below:
  (5) $$\begin{array}{c} t_{n+1}=\left[1+b\left(t_{n-1}-c\right)+379R_{n}^{2}\right]\left(\mathrm{mod}\;1\right), \end{array}$$
  where *b* and *c* are Henon map parameters that are specified by *b* = 0.3 and 1.07 ≤ *c* ≤ 1.09. Chaotic maps are constrained within the limit by performing modulo operation (mod⁡  1). In addition, such operation retains the sequence convergence. The generated sequence is real numbers and hence it is quantized into binary system using simple threshold as below:
  (6) $$\begin{array}{c} c\left(n\right)=\{\begin{array}{cc} 1, & t_{n}\geq 0.5,\\ 0, & t_{n}<0.5. \end{array} \end{array}$$
(vii) Finally, the binary sequence *c*(*n*) generated above is used to encrypt the generated authentication code. The authentication code is produced by feeding the Quran image into hash function; the authentication code is defined in binary systems as *h*(*n*) ∈ {0, 1}. Then, using equation below the encrypted authentication code is attained:
  (7) $$\begin{array}{c} l\left(n\right)=\overset{N}{\underset{n=1}{\sum }}h\left(n\right)⊕c\left(n\right), \end{array}$$
  where *N* is length of the authentication code.

The decryption of authentication code is simply an opposite procedure of encryption process as explained above. Using same secret keys, the decryption is using the following formula:

(8) $$\begin{array}{c} h^{\prime }\left(n\right)=\overset{N}{\underset{n=1}{\sum }}l^{\prime }\left(n\right)⊕c\left(n\right). \end{array}$$

### 3.2. The Proposed Watermark Embedding Process

The encrypted authentication code is embedded into the host image according to steps below.

(i) Firstly, the host image is brought into wavelet domain by performing 2D discrete wavelet transforms using Daubechies function. The decomposition generates *L* resolution levels.
(ii) Decomposition process using DWT will produce four matrices defined as wavelet coefficients, *LH*_*p*_, *HL*_*p*_, and *HH*_*p*_. The resolution level is determined with *p* × *L*. In this paper, the decomposition level of the *p* = 1 is considered. According to Run et al. [24], selecting higher *p* value to produce higher subbands level tolerates a greater tampering detection, but it decreases the localization accuracy against image tampering.
(iii) After decomposition, one decompose matrix called wavelet coefficients *CW*  are rounded into closest integer. Then, the matrix is fragmented into small size of nonoverlapping block. Such fragmented blocks ensure that matrix of wavelet coefficients parallel to the same spatial spot will be inserted with watermark code. The payload of watermark code in a block is controlled using *n* parameter. The block size should be determined properly to allow sufficient watermark payload and to maintain fine image quality. The suitable block size also ensures optimum localization capability on tampered region. Equation below is used to calculate the block size *b*:
  (9) $$\begin{array}{c} b\left(n\right)=\left\lceil \sqrt{2^{n}}\right\rceil . \end{array}$$
(iv) The watermark information that is already prepared as described in Section 3.1 is utilized. Such watermark resists local attacks problem because the information has been blurred using chaotic map [24]. The bits of watermark are defined as *l*_*m*_. It is known that the watermark length of *l*_*m*_ is influenced by the selected hash function. In this regard, simple function is required to select the bits sequentially with respect to *n* parameter (*n* value is same as in step (iii)) as defined below:
  (10) $$\begin{array}{c} W=\{\begin{array}{cc} {\text{LSBit}}_{1\ldots n}, & n\leq 8,\\ {\text{LSBit}}_{1\ldots 8}, & n>8. \end{array} \end{array}$$
(v) The selected authentication bits *W* are hidden into the wavelet coefficients using weighted-sum function, *f*(*x*) [17]. The block of wavelet coefficients is fed into weighted-function and then the resulting value is compared with selected authentication bits; both values are treated in decimal form. A loop is performed until the weighted-sum value is equal to the selected authentication bits. The value in a block *B* of wavelet coefficient is modified ±1 with respect to function as follows:
  (11) $$\begin{array}{c} S=W-f\left(x\right),\\ B\left(d\right)=\{\begin{array}{cc} B_{\left|s\right|}+1, & 0<\left|S\right|\leq 2^{n},S>0\\ B_{\left|s\right|}-1, & 0<\left|S\right|\leq 2^{n},S<0\\ B_{\left|s-2^{m+1}\right|}+1, & 2^{n}<\left|S\right|\leq 0,S>0\\ B_{\left|s-2^{m+1}\right|}-1, & 2^{n}<\left|S\right|\leq 0,S<0. \end{array} \end{array}$$
(vi) After processing all blocks of the wavelet coefficients, the new wavelet coefficients are stored as *CW*_1_ *'*. Afterward, *CW*_1_ *'* along with three decomposition matrixes (*LH*, *HL*, and *HH*) are inverted back into spatial domain using 2D-Inverse DWT. The new image *I*_1_ *'* is decomposed using 2D-DWT to obtain the wavelet coefficient, stored as *CW*_2_. Using equation below matrix *D* is calculated:
  (12) $$\begin{array}{c} D=CW_{1}^{\prime }-CW_{1}^{\prime \prime }. \end{array}$$
(vii) The matrix *D* as generated above consists only of three kinds of values {−1, 0, + 1}. Since matrix *D* is halftone of the new image size then it requires stretching the matrix such that it has the same size of the image size. Such aim can be done by multiplying the matrix size by two and filling the gap with zero value. Further, the stretched matrix *D* is embedded into spatial domain of new image *I*_2_ by altering the least significant bits of its pixels with value in matrix *D*.

### 3.3. The Watermark Authentication Process

The authentication process is allowed by tamper localization on the protected image if present. This process can be seen as reverse of the embedding process. The following steps describe the authentication process.

- As explained above, two watermarks are embedded separately on wavelet domain and then on spatial domain. It requires extracting them in reverse way. In spatial domain, the watermarks are hidden in least significant bits of pixels with respect to the matrix *D*. In this regard, the LSB of each pixel is extracted by taking the two insignificant bits; the value is then saved in matrix *E*.
- Matrix *E* must be suppressed into half size because it contains gap values of zeros. The new image after removing the insignificant bits is stored as *I*_*e*_.
- The new image *I*_*e*_ is decomposed into wavelet domain using 2D-DWT. It decomposes into first level subbands same as on watermarking process. Hence, it generates first wavelet coefficient, *LH*_1_, *HL*_1_, and *HH*_1_. The generated wavelet coefficient *CW*_1_ is floored into nearest integer. Afterward, *CW*_1_ is added with matrix *E* to generate new wavelet coefficient *CW*_2_.
- The *CW*_2_ is processed block-by-block with block size same as defined during watermarking process. The weighted-sum function is utilized to obtain weight value of the block. The weight value is decrypted prior to comparing with authentication bits that are stored in database. The valid block should have same value with the selected authentication bits; otherwise it is categorized as tampered block. The wavelet coefficient value of tampered block is modified into zero values to localize the tamper region. Finally, authenticated image is generated by transforming the wavelet coefficient using 2D-DWT. A black box will exist if any forgery is present.

## 4. Implementation and Experimental Results

**Figure 3:**
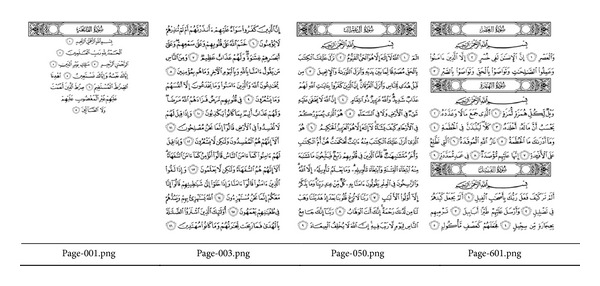
Some Quran images in the dataset.

The performance of the proposed method is assessed with two datasets taken from popular android applications. Each one of the datasets consists of 604 images of the Quran pages. The file formats for the datasets A and B are JPEG and PNG, respectively. Dataset A has a border for each page and the color defined in RGB spaces. Dataset B does not have a border and is presented in the indexed grayscale image.  Figure 3 presents some Quran images and Table 1 detailed the characteristics of each dataset.

The experiment is conducted based on the common methodology as reported throughout the literature [15–20, 25]. The assessment includes localization accuracy against image forgery and measures image quality after watermarking process. The result is presented for four pages only, selected from pages 1, 3, 50, and 601, for the sake of simplicity of the presentation. These four pages are considered because they represent the whole Quran pages. Page 1 consists only of one chapter and it is like page 50. Page 3 is almost similar with all pages of the Holy Quran. Page 50 shows the beginning of a new chapter and page 601 consists of several chapters in the page. The main concern of fragile watermarking method is the ability to detect the manipulation while preserving the host image after watermarking. Hence, several methods of image quality measurement are utilized to determine the quality of the watermarked image. The image quality methods are as in Table 2.

The image quality after the watermarking process is measured using the above-mentioned equations. In the literature, some methods have reported the reference value that indicates adequate image quality. The known acceptable values are shown in Table 3.

Proposed fragile watermarking method should be capable of detecting any manipulation and locate the tampered location. Certain form of manipulation can be unintended; for example, the usage of the compression schema and other manipulations can be intended including resizing, cropping, rotation, and other image manipulations. Those image manipulations can be summarized as follows.

- Lossy compression such as JPEG and MPEG practically damages the image quality through irrecoverable loss of information.
- Geometric distortions alter the image symmetry and include operations such as cropping, rotations, scaling, and translation.
- General signal processing procedures are such as image filtering, resampling, color reduction, local conversation of pixels, and adding of constant offset to the pixel values.
- Other intentional attacks are done by human hand, for example, performing watermarking on watermarked image or scanning watermarked image to produce same image that bypassed the watermark information.

As summarized above, six common image manipulations that covered the above-mentioned image manipulations are considered to demonstrate the performance of the proposed approach. The considered manipulations are only applicable for Quran pages, which should not alter the content extreme (e.g., rotation or flip the image). The considered image manipulations applied on the dataset are presented as in Table 4.

Please note that the pixel manipulation is too small. Therefore, it is hard to see by using naked eye. Then, replacement manipulation is executed by exchanging one verse of watermarked Quran image with another verse. It is clear that such manipulation will distort the content. Such replacement is a challenge to the nonprofessional to be discovered because of the lack of knowledge regarding the authentic Quran. The five image manipulations (except JPEG compression) that are applied on Quran image page number 3 are presented in Table 5. As mentioned in Section 3, the proposed method requires a parameter for the block size, defined as *n*. The experiment will be conducted for a range of *n* values, where *n* = {1,2,…, 15}. Each of *n* values is subjected to the above-mentioned image manipulations.

**Figure 4:**
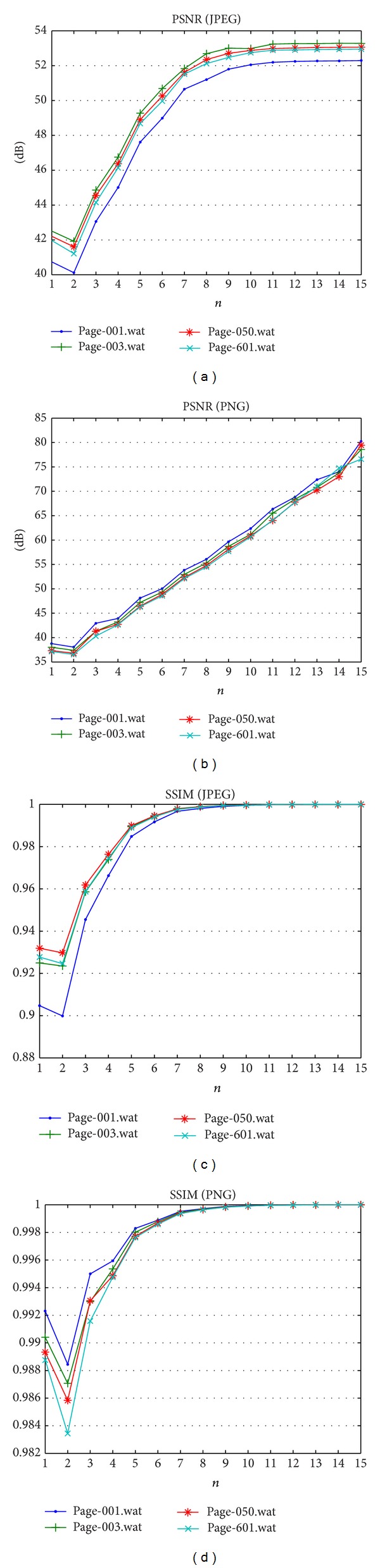
PSNR and SSIM results of watermarked image.

Initially, the experiment is carried out to evaluate the PSNR value of watermarked image, because this metrics is commonly used and suitable to measure the performance of digital watermarking method. The proposed method can achieve PSNR value above 42 dB [3, 17] after *n* > 3 as shown in Table 6 and Figure 4, for both datasets A and B. It confirms that the level of image distortion following watermarking process is low enough even when considering a small *n* value. The first important feature of the fragile watermark is imperceptibility. In order to discover the difference between the watermarked image and original image, hence the SSIM metric can be utilized to inspect the visual similarity [57]. SSIM has proved to be in line with the human visual system and is able to evaluate the relationship of the two different images, including image contrast, image brightness, and three aspects in the image structure. The SSIM value during experiment shows near to one when *n* > 8, as depicted in Table 7 and Figure 4. Hence, the proposed method is able to preserve the image quality positively.

Afterward, the experiment is conducted by performing the dewatermarking process minus any image attacks to verify whether the authentication process can be accomplished properly or not. The result indicates that the watermarked image can pass the authentication perfectly for every *n* value to the parameter of proposed method. The *n* parameter is ranging from 1 to 15. The percentage of authentic blocks calculated by dividing the authenticated blocks with overall quantity of all blocks is 100% for both datasets A and B. It can be concluded that the proposed method can perform embedding and extracting of the watermark on host image perfectly.

**Figure 5:**
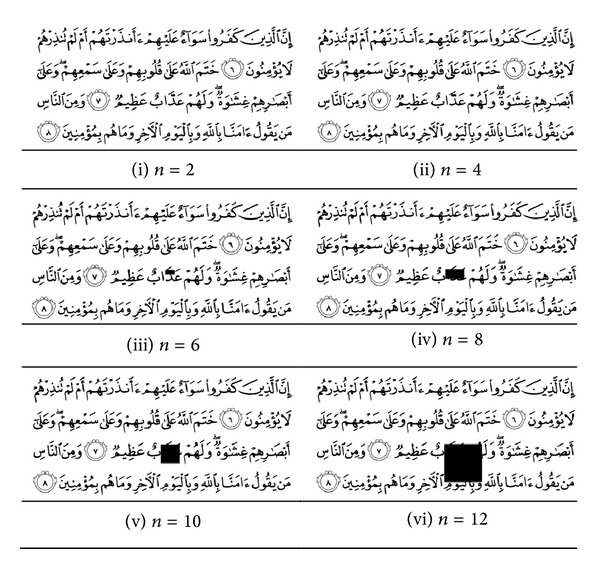
Tamper detection on pixel attack (page-003.png).

**Figure 6:**
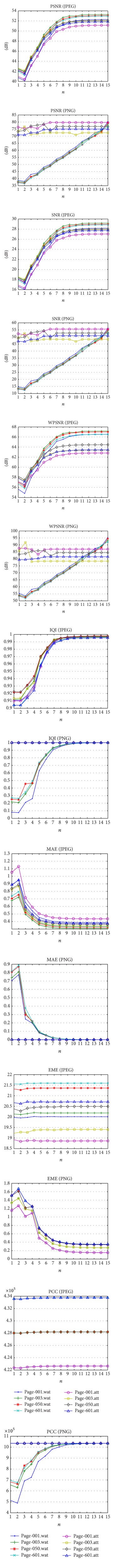
Image quality metrics on pixel manipulation attack.

**Figure 7:**
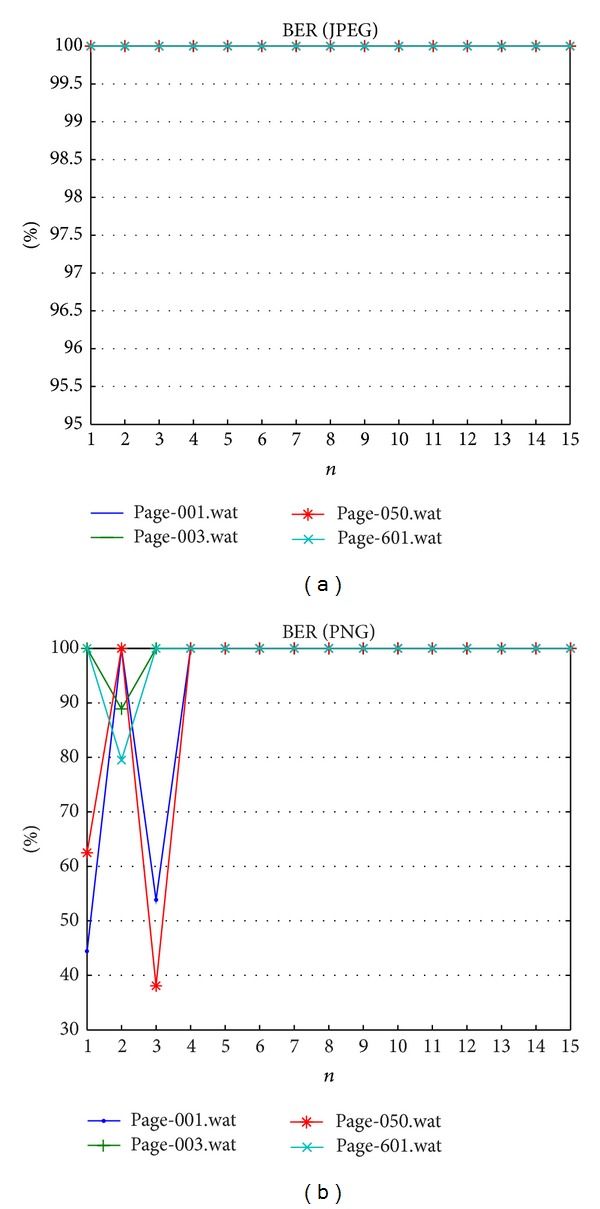
Bit error rates result against pixel manipulation attack.

The next evaluation is carried out to study the capability of proposed method with respect to the pixel manipulation as reported in Table 5. Table 5 of the pixel manipulation shows a particular region of the watermarked Quran image that has been altered, which is a single dot of the verse deleted. Such manipulation undoubtedly annoys the integrity of the Quran's content as the dot(s) has significance in Arabic alphabets. The parameter *n* value within range {1, 15} is tested to understand the influence against tamper detection. Tables 8 and 9 show PSNR and BER results. Figures 5(i)–5(vi) show the location of the tampered region in the image as black box. Image quality metrics confirm that after attack the quality is decreased and tends to have a constant value as depicted in Figure 6. The fragility reported in Figure 7 that shows on JPEG dataset pixel manipulation can be detected under any *n* value. Meanwhile, fragility on PNG dataset can achieve 100% only after *n* ≥ 5.

**Figure 8:**
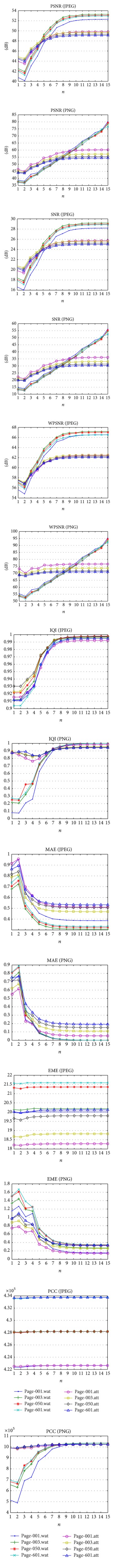
Image quality metrics on Gaussian blurring attack.

**Figure 9:**
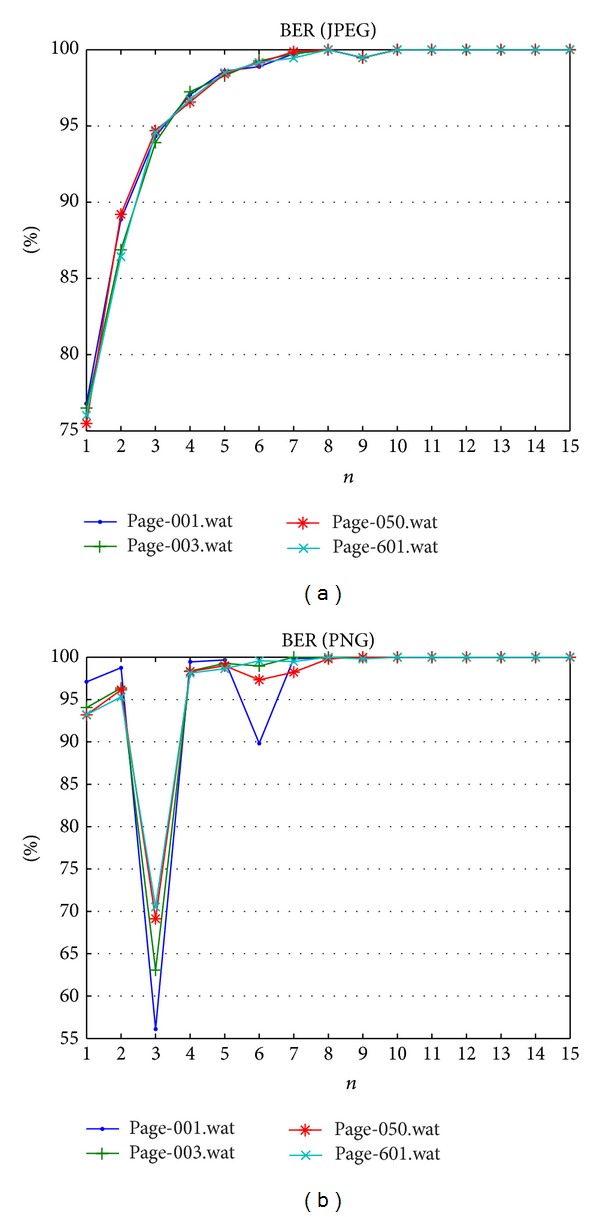
Bit error rates result against Gaussian blurring attack.

The Gaussian filter is applied on the watermarked image, and the fragility is measured. The filter is blurring the Quran image that makes some verses unreadable or vague.  Table 10 shows the image quality measurement to indicate the image quality after attack and Table 11 reports that the BER results indicated how fragile the proposed method is. As expected, changes in the magnitude of gradient vectors due to Gaussian filter can be detected properly. Hence, the proposed method is fragile to blurring attack. Blurring attack has decreased the image quality as reported in Figure 8, the PSNR result and other metrics shows obtain lower values compare with metrics' values before apply blurring attack. BER for both datasets are shown in Figure 9 and finest fragility can be achieved only after *n* ≥ 8.

**Figure 10:**
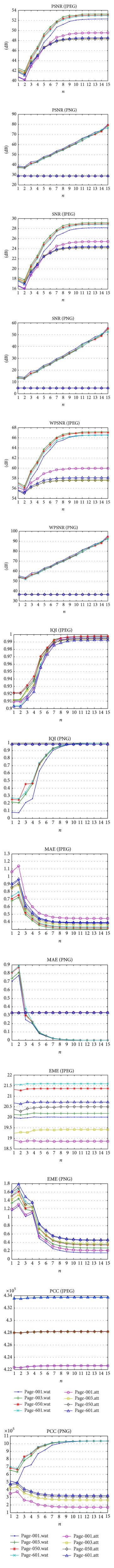
Image quality metrics on Gaussian noise attack.

**Figure 11:**
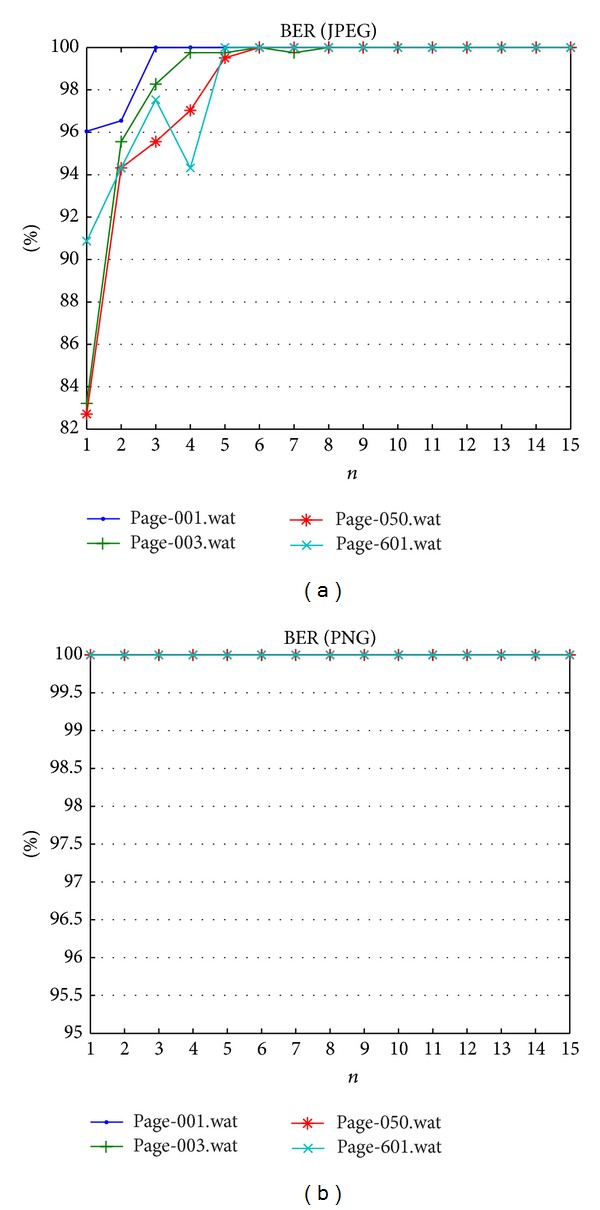
Bit error rate result against Gaussian noise attack.

The Quran image should not contain any noise because a dot is a matter in Arabic letters. Hence, a Gaussian noise that is commonly used to add noises in image processing is considered. The Gaussian noise does not have structure and it is difficult to eliminate such noises without suffering modifications to the image itself. Tables 12 and 13 show the PSNR and BER results of the proposed method, respectively, after Gaussian noise is inserted to the watermarked images. The result confirms that the proposed fragile watermarking method is capable of localizing the noise attack. Image quality is decreased after this attack as presented in Figure 10.  Figure 11 reported fragility on PNG dataset better than JPEG dataset. Fragility on JPEG dataset is superior after *n* > 6.

**Figure 12:**
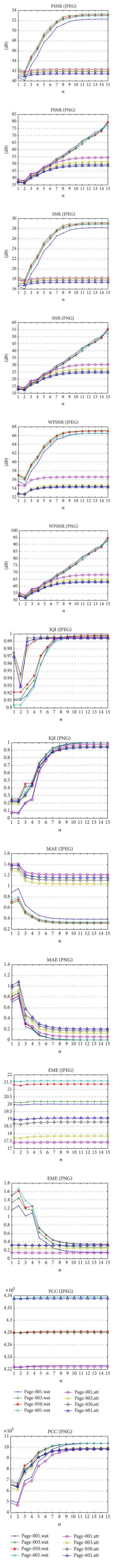
Image quality metrics on median filtering attack.

**Figure 13:**
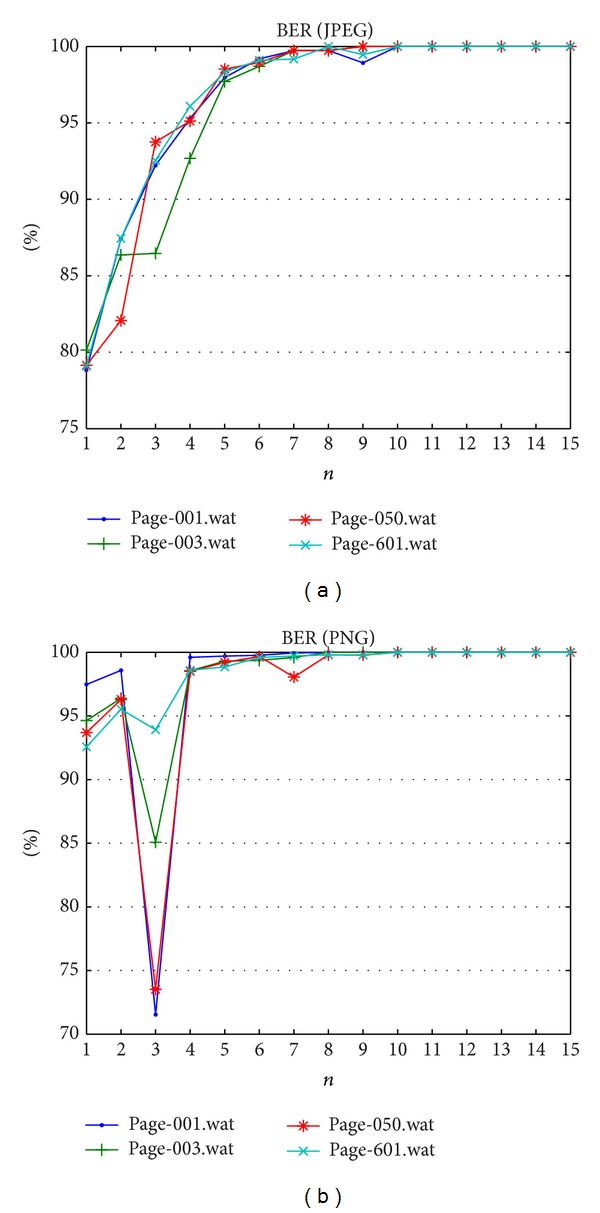
Bit error rate result against median filtering attack.

The succeeding considered attack is median filtering attack. Such attack is known as nonlinear attack and alters the gradient vector of image much more than Gaussian filter. The median filter does not change the edge points unlike Gaussian filter. The PSNR and the BER results of proposed method under median filtering attacks are presented in Tables 14 and 15, respectively. As mentioned before, the median filters are executed with 3 × 3 window. The result demonstrates that tamper localization after a median filtering attack is very efficient in both dataset A and dataset B. This attack degrades the image quality more than previous attacks as presented in Figure 12. The proposed method can attain acceptable fragility only after *n* ≥ 8 for both datasets as depicted in Figure 13.

**Figure 14:**
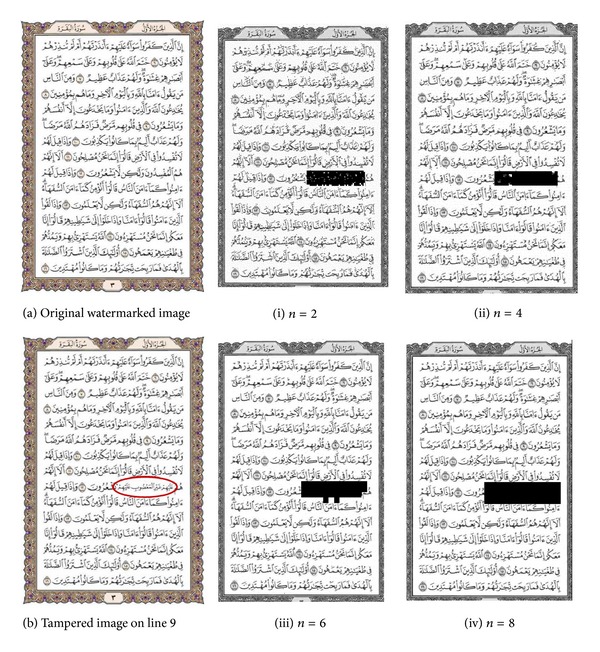
Tamper detection on collage attack (page-003.jpg).

**Figure 16:**
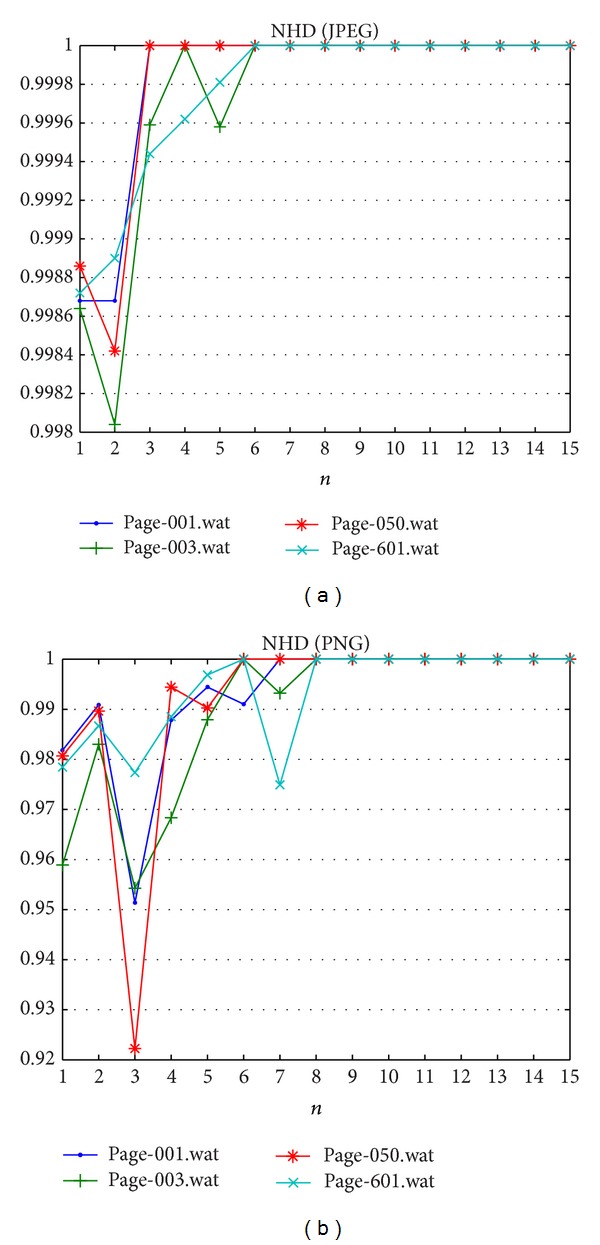
NHD result on collage attack, *n* = [1,15].

The collage attack is one of the security issues on the fragile watermarking method that needs to be concerned. In this regard, the watermarked image is altered by replacing the verse line number 9 with another verse. The pasted versed is taken from watermarked image and hence it has authentication code. Figures 14(i)–14(iv) and 15(i)–15(iv) report the position of the altered region in both datasets as black box. The result demonstrates even with small *n* value the altered region can be detected but not accurately. The proposed method can localize the collage regions correctly when the parameter *n* > 4. The NHD metric is utilized to estimate the localization performance by considering the modification between watermarked image and attacked image. The NHD is shown in Figure 16; the values expressing the proposed method can detect the collage attack as long as the *n* parameter is greater than or equal to 8.

**Figure 17:**
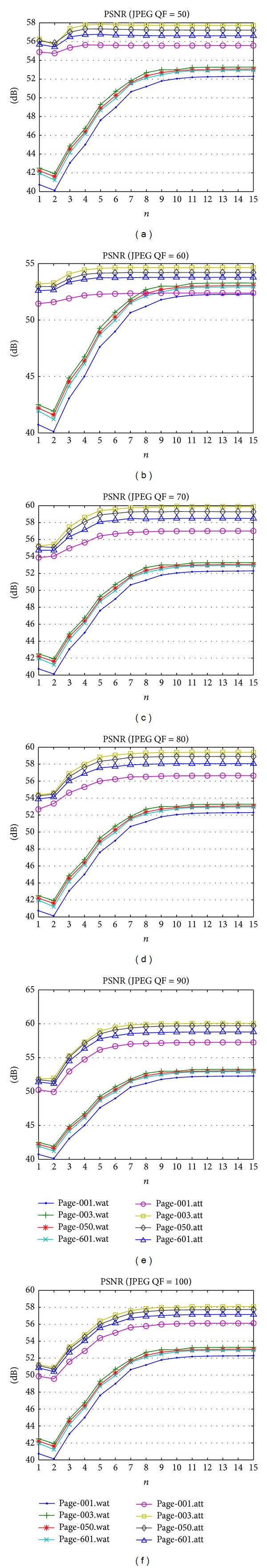
PSNR before and after JPEG compression, QF = [50,100].

**Figure 18:**
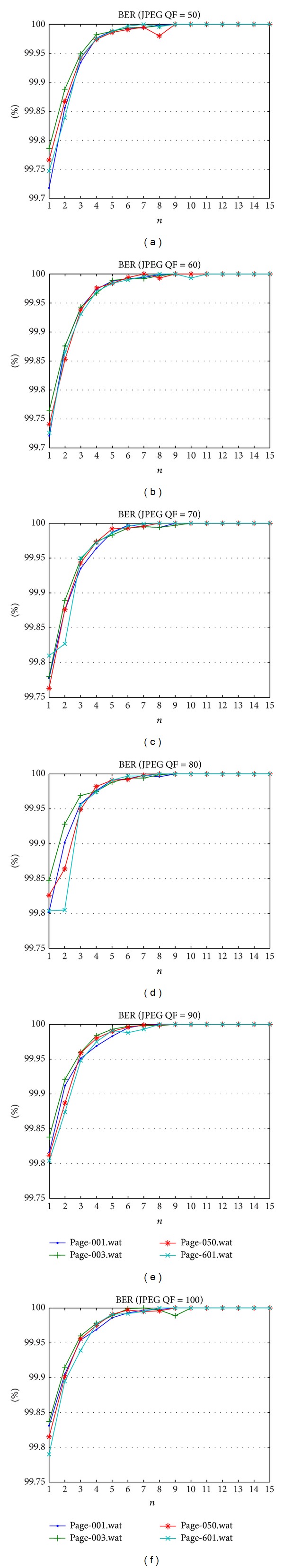
Bit error rate result against JPEG compression, QF = [50,100].

Finally, JPEG compression is tested on proposed method to evaluate the fragility against nonmalicious attack. The evaluation is considered from soft to modest JPEG compression with quality factor (QF) of JPEG compression within range {50, 100}. PSNR results are presented in Figure 17; the compression does not affect the image quality. The BER of the watermarked image following authentication process is measured.  Figure 18 reported the BER results for *n* = 1 to *n* = 15. As expected, the BER value is high which means proposed method is fragile to JPEG compression with respect to any *n* value. Such fragility is contributed from embedding process that considers the coefficients matrix of the first wavelet subbands. The first wavelet subbands are altered during the JPEG compression. A proper value for *n* parameters can vary from one application to another. Nevertheless, it is suggested that *n* value equal to eight (8) is applicable for general digital watermarking purposes.

## 5. Conclusion and Future Work

Based on the literature review, authors found no study attempt to explore the digital watermarking for the Holy Quran images. Most of the recent digital watermarking methods are applied on natural images, for example, Lena, baboon, and other popular images. Hence, there are a gap and an open issue related to preserving the integrity of the Holy Quran. This paper brings novel issue to the society and presents one of the solutions to protect the Holy Quran from common image tampering. The proposed method is intended as fragile watermarking that considers both wavelet domain and spatial domain. The first layer of watermarking method is applied on wavelet domain and second layer on spatial domain. The host image is transformed into wavelet domain using discrete wavelet transformation (DWT) prior to hiding the watermark. The recent methods demonstrated that such watermarking schema in the wavelet domain is robust to brute-force attack rather than watermarking method in the spatial domain. Furthermore, proposed method can be categorized as a block-based approach and it introduces correlation between the blocks that make resistance to collage attack. The two-layer watermarks also guarantee challenging the intruder to bypass the authentication without breaking the watermarked image. Thanks are due to chaotic maps that are very sensitive to initial value, demonstrate greatly blurred authentication code, and protect the watermark against local attacks. According to the result of image quality metrics, the proposed method obtained satisfactory image quality after embedding the procedure. Finally, several image attacks that applied into the watermarked image demonstrated that the proposed method yields promising localization of the image tampering at a minimum watermark payload.

In the future, analysis on other attacks can be considered such as blurring attack, noise attack, and other known attacks. Further, the study should work on various image formats, for example, JPEG2000, TIFF or BMP formats. Investigation on the image quality after watermarking process, using metrics other than PSNR and SSIM, is important. Implementing watermarks on higher level of decomposition can also be explored. Finally, the security can also be improved by improving the encryption part.

## Figures and Tables

**Figure 15:**
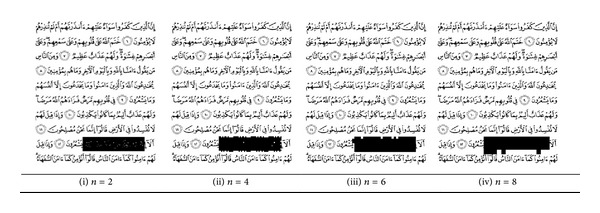
Tamper detection on collage attack (page-003.png).

**Table 1:** Characteristics of the datasets.

	Dataset A	Dataset B
Source	Quran Kareem [10]	Quran Android [10]
Format	JPEG	PNG
Image compression	Lossy	Lossless
Dimensions (width × height)	547 × 793	800 × 1294
Number of pages	604	604
Min. file size	67 KB	30 KB
Max. file size	78 KB	100 KB
Total dataset file size	42.6 MB	51.1 MB
Border on each page	Present	No border
Color image	Yes (full color)	Grayscale (limited)

**Table 2:** Common image quality metrics.

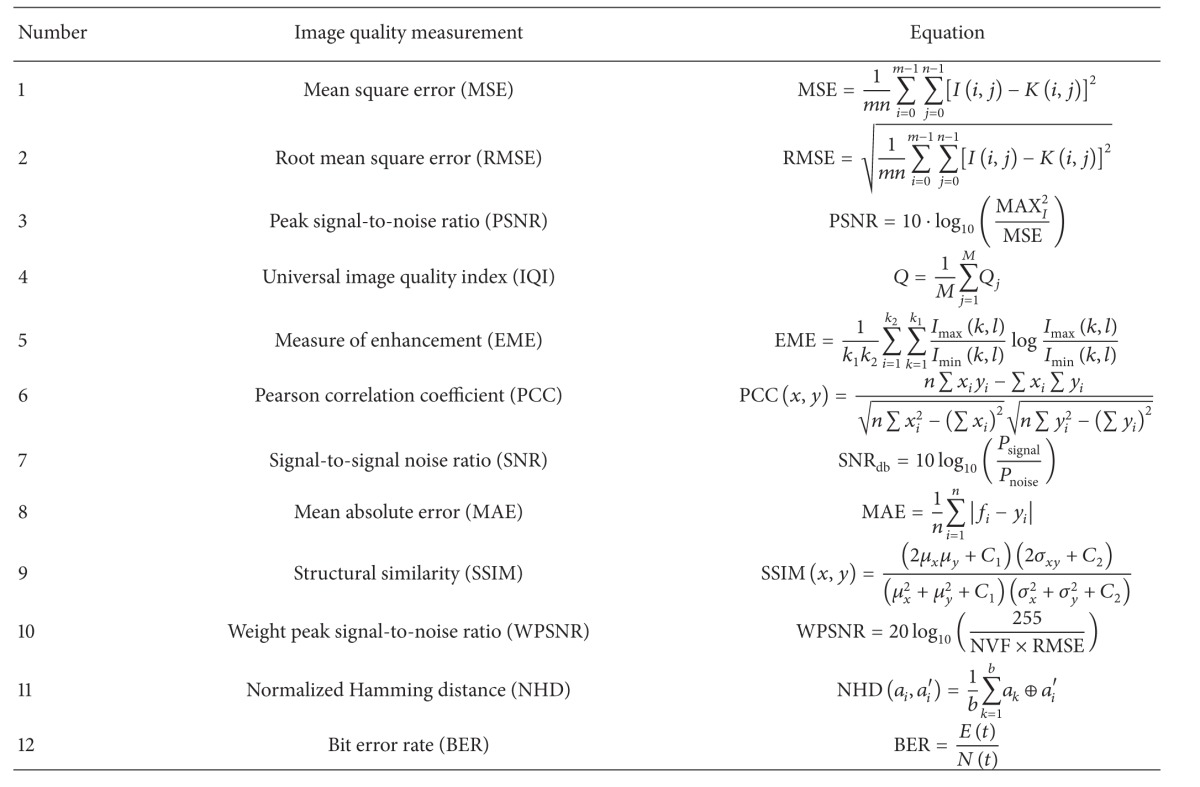

**Table 3:** Known acceptable value in image quality measurement.

Number	Image quality measurement	Known acceptable value
1	Peak signal-to-noise ratio (PSNR)	≥42 dB, greater than or equal to 42 db is better
2	Universal image quality index	~1.0, near to 1.0 is perfect
3	Signal-to-signal noise ratio (SNR)	≥20 dB, greater than or equal to 20 db is better
4	Structural similarity (SSIM)	~1.0000, near to 1.0000 is perfect
5	Normalized Hamming distance (NHD)	=1, equal to 1 is perfect

**Table 4:** Six image manipulations applied on datasets.

Number	Image manipulation	Parameter	JPEG	PNG
1	Pixel manipulation	Window size 10 × 10	*✓*	*✓*
2	Blurring	Gaussian filter, sigma = 0.5	*✓*	*✓*
3	Noise	Gaussian noise, 150 blocks, block size 3 × 3, mean = 0.4, variance = 0.01	*✓*	*✓*
4	Median filtering	Filter size 3 × 3	*✓*	*✓*
5	Replacement/collage	—	*✓*	*✓*
6	JPEG compression	Quality factor {50, 100}	*✓*	*✗*

**Table 5:** Examples of image manipulations on page-050.png.

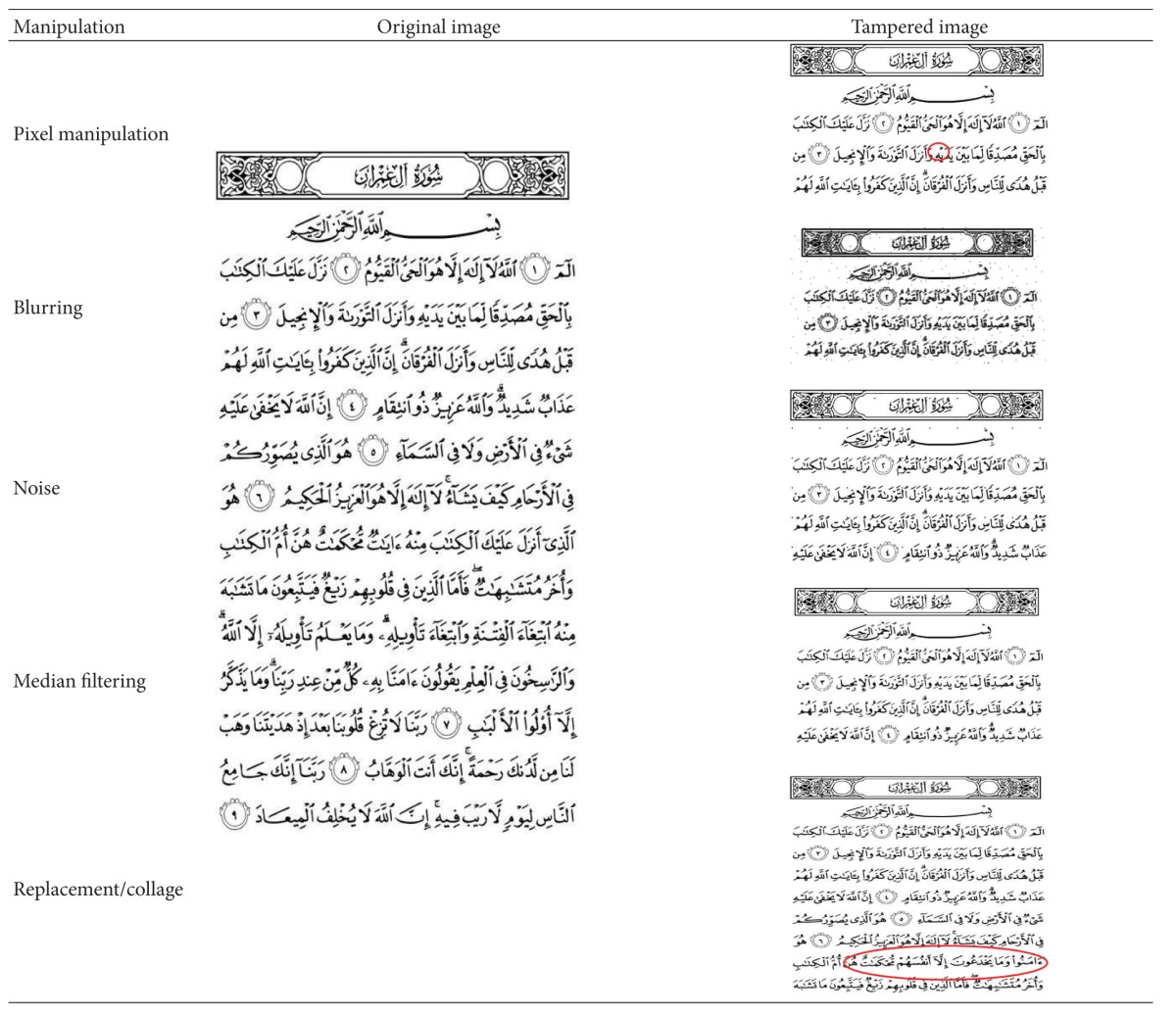

**Table 6:** PSNR results of proposed watermarking method.

#	1	2	3	4	5	6	7	8	9	10	11	12	13	14	15
JPEG															
001	40.74	40.12	**43.06**	45.01	47.61	48.99	50.65	51.20	51.80	52.05	52.19	52.25	52.27	52.28	52.30
003	42.50	41.93	**44.86**	46.75	49.27	50.69	51.84	52.69	53.00	52.99	53.24	53.27	53.27	53.29	53.28
050	42.20	41.61	**44.55**	46.37	48.90	50.26	51.62	52.35	52.71	52.88	52.98	53.01	53.04	53.06	53.06
601	41.97	41.22	**44.15**	46.14	48.68	49.97	51.52	52.12	52.48	52.75	52.89	52.90	52.93	52.94	52.95

PNG															
001	38.78	38.05	**42.94**	43.94	48.10	50.04	53.84	56.05	59.64	62.35	66.37	68.80	72.36	74.01	80.24
003	38.02	37.39	41.30	**43.22**	47.20	49.35	52.95	55.29	58.69	61.17	65.52	68.35	70.81	73.75	78.57
050	37.34	36.81	41.30	**42.74**	46.43	48.89	52.32	54.77	58.10	60.86	64.01	67.77	70.21	73.00	79.44
601	37.15	36.55	40.31	**42.64**	46.30	48.58	52.12	54.49	57.70	60.66	64.11	67.71	70.96	74.78	76.58

**Table 7:** SSIM results of proposed watermarking method.

#	1	2	3	4	5	6	7	8	9	10	11	12	13	14	15
JPEG															
001	0.905	0.900	0.946	0.966	0.985	0.992	0.997	0.998	0.999	**1.000**	1.000	1.000	1.000	1.000	1.000
003	0.925	0.924	0.959	0.974	0.990	0.994	0.998	0.999	**1.000**	1.000	1.000	1.000	1.000	1.000	1.000
050	0.932	0.930	0.962	0.976	0.990	0.995	0.998	0.999	0.999	**1.000**	1.000	1.000	1.000	1.000	1.000
601	0.928	0.925	0.959	0.974	0.989	0.994	0.998	0.999	0.999	**1.000**	1.000	1.000	1.000	1.000	1.000

PNG															
001	0.992	0.988	0.995	0.996	0.998	0.999	**1.000**	1.000	1.000	1.000	1.000	1.000	1.000	1.000	1.000
003	0.990	0.987	0.993	0.995	0.998	0.999	0.999	**1.000**	1.000	1.000	1.000	1.000	1.000	1.000	1.000
050	0.989	0.986	0.993	0.995	0.998	0.999	0.999	**1.000**	1.000	1.000	1.000	1.000	1.000	1.000	1.000
601	0.989	0.983	0.992	0.995	0.998	0.999	0.999	**1.000**	1.000	1.000	1.000	1.000	1.000	1.000	1.000

**Table 8:** PSNR result before and after pixel manipulation.

#		1	2	3	4	5	6	7	8	9	10	11	12	13	14	15
JPEG																
001	Bef.	40.74	40.12	43.06	45.01	47.61	48.99	50.65	51.20	51.80	52.05	52.19	52.25	52.27	52.28	52.30
	Aft.	40.98	40.40	43.16	44.98	47.33	48.53	49.89	50.33	50.79	50.97	51.07	51.11	51.13	51.13	51.15
003	Bef.	42.50	41.93	44.86	46.75	49.27	50.69	51.84	52.69	53.00	52.99	53.24	53.27	53.27	53.29	53.28
	Aft.	42.67	42.13	44.91	46.64	48.87	50.06	50.95	51.59	51.81	51.81	51.98	52.00	52.00	52.01	52.01
050	Bef.	42.20	41.61	44.55	46.37	48.90	50.26	51.62	52.35	52.71	52.88	52.98	53.01	53.04	53.06	53.06
	Aft.	42.42	41.87	44.66	46.38	48.68	49.86	50.97	51.55	51.82	51.95	52.04	52.06	52.08	52.09	52.09
601	Bef.	41.97	41.22	44.15	46.14	48.68	49.97	51.52	52.12	52.48	52.75	52.89	52.90	52.93	52.94	52.95
	Aft.	42.19	41.47	44.25	46.09	48.39	49.49	50.75	51.21	51.48	51.68	51.78	51.79	51.81	51.82	51.82

PNG																
001	Bef.	38.78	38.05	42.94	43.94	48.10	50.04	53.84	56.05	59.64	62.35	66.37	68.80	72.36	74.01	80.24
	Aft.	76.38	75.57	76.50	75.44	78.33	79.67	79.67	79.67	79.67	79.67	79.67	79.67	79.67	79.67	79.67
003	Bef.	38.02	37.39	41.30	43.22	47.20	49.35	52.95	55.29	58.69	61.17	65.52	68.35	70.81	73.75	78.57
	Aft.	73.43	76.73	71.22	71.60	72.42	72.42	72.42	72.42	72.42	70.92	72.42	72.42	72.42	72.42	72.42
050	Bef.	37.34	36.81	41.30	42.74	46.43	48.89	52.32	54.77	58.10	60.86	64.01	67.77	70.21	73.00	79.44
	Aft.	73.31	74.07	77.13	77.80	78.51	73.47	76.90	76.90	76.90	76.90	76.90	76.90	76.90	76.90	76.90
601	Bef.	37.15	36.55	40.31	42.64	46.30	48.58	52.12	54.49	57.70	60.66	64.11	67.71	70.96	74.78	76.58
	Aft.	71.00	70.84	72.44	72.44	74.74	74.69	75.02	75.02	75.02	75.02	75.02	75.02	75.02	75.02	75.02

**Table 9:** Bit error rates of proposed method.

#	1	2	3	4	5	6	7	8	9	10	11	12	13	14	15
001	100	100	100	100	100	100	100	100	100	100	100	100	100	100	100
003	100	100	100	100	100	100	100	100	100	100	100	100	100	100	100
050	100	100	100	100	100	100	100	100	100	100	100	100	100	100	100
601	100	100	100	100	100	100	100	100	100	100	100	100	100	100	100
001	44	100	53.8	100	100	100	100	100	100	100	100	100	100	100	100
003	100	88.9	100	100	100	100	100	100	100	100	100	100	100	100	100
050	63	100	38.1	100	100	100	100	100	100	100	100	100	100	100	100
601	100	79.5	100	100	100	100	100	100	100	100	100	100	100	100	100

**Table 10:** PSNR result on Gaussian blurring.

#		1	2	3	4	5	6	7	8	9	10	11	12	13	14	15
JPEG																
001	Bef.	40.74	40.12	43.06	45.01	47.61	48.99	50.65	51.20	51.80	52.05	52.19	52.25	52.27	52.28	52.30
	Aft.	43.91	43.49	45.62	46.85	48.24	48.82	49.39	49.54	49.70	49.76	49.80	49.81	49.81	49.82	49.82
003	Bef.	42.50	41.93	44.86	46.75	49.27	50.69	51.84	52.69	53.00	52.99	53.24	53.27	53.27	53.29	53.28
	Aft.	44.85	44.49	46.49	47.54	48.64	49.08	49.39	49.58	49.64	49.64	49.68	49.69	49.69	49.69	49.69
050	Bef.	42.20	41.61	44.55	46.37	48.90	50.26	51.62	52.35	52.71	52.88	52.98	53.01	53.04	53.06	53.06
	Aft.	44.59	44.21	46.17	47.17	48.23	48.69	49.04	49.20	49.27	49.30	49.32	49.32	49.33	49.33	49.33
601	Bef.	41.97	41.22	44.15	46.14	48.68	49.97	51.52	52.12	52.48	52.75	52.89	52.90	52.93	52.94	52.95
	Aft.	44.42	43.92	45.85	46.94	48.02	48.42	48.82	48.94	49.00	49.06	49.08	49.08	49.09	49.09	49.09

PNG																
001	Bef.	38.78	38.05	42.94	43.94	48.10	50.04	53.84	56.05	59.64	62.35	66.37	68.80	72.36	74.01	80.24
	Aft.	46.33	44.92	49.87	50.55	54.47	55.40	57.63	58.44	59.43	59.74	60.03	60.13	60.18	60.20	60.22
003	Bef.	38.02	37.39	41.30	43.22	47.20	49.35	52.95	55.29	58.69	61.17	65.52	68.35	70.81	73.75	78.57
	Aft.	45.39	44.32	48.04	49.64	52.80	53.91	55.47	56.12	56.68	56.90	57.10	57.15	57.17	57.19	57.20
050	Bef.	37.34	36.81	41.30	42.74	46.43	48.89	52.32	54.77	58.10	60.86	64.01	67.77	70.21	73.00	79.44
	Aft.	44.18	44.01	47.91	49.11	51.49	52.88	54.08	54.63	55.04	55.22	55.34	55.40	55.42	55.44	55.45
601	Bef.	37.15	36.55	40.31	42.64	46.30	48.58	52.12	54.49	57.70	60.66	64.11	67.71	70.96	74.78	76.58
	Aft.	43.94	43.63	46.92	48.77	50.96	52.14	53.25	53.70	54.04	54.21	54.31	54.35	54.37	54.38	54.39

**Table 11:** Bit error rates on Gaussian blurring.

#	1	2	3	4	5	6	7	8	9	10	11	12	13	14	15
001	77	88.9	94.3	97	98.6	98.9	99.7	100	99.5	100	100	100	100	100	100
003	76	86.9	93.9	97.2	98.3	99.3	99.7	100	99.5	100	100	100	100	100	100
050	75	89.2	94.7	96.6	98.4	99.1	99.9	100	99.5	100	100	100	100	100	100
601	76	86.4	94.6	96.7	98.5	99.2	99.4	100	99.5	100	100	100	100	100	100
001	97	98.7	56.1	99.4	99.7	89.8	99.8	99.9	100	100	100	100	100	100	100
003	94	96.4	63	98.4	99.3	99	100	100	100	100	100	100	100	100	100
050	93	96.1	69.1	98.3	99	97.3	98.2	99.8	100	100	100	100	100	100	100
601	93	95.3	70.5	98.1	98.6	99.6	99.5	100	99.8	100	100	100	100	100	100

**Table 12:** PSNR results on Gaussian noise attack.

#		1	2	3	4	5	6	7	8	9	10	11	12	13	14	15
JPEG																
001	Bef.	40.74	40.12	43.06	45.01	47.61	48.99	50.65	51.20	51.80	52.05	52.19	52.25	52.27	52.28	52.30
	Aft.	40.80	40.25	42.87	44.53	46.59	47.59	48.64	48.97	49.29	49.42	49.49	49.52	49.53	49.54	49.55
003	Bef.	42.50	41.93	44.86	46.75	49.27	50.69	51.84	52.69	53.00	52.99	53.24	53.27	53.27	53.29	53.28
	Aft.	42.07	41.60	43.92	45.24	46.73	47.42	47.88	48.19	48.29	48.28	48.36	48.37	48.37	48.37	48.37
050	Bef.	42.20	41.61	44.55	46.37	48.90	50.26	51.62	52.35	52.71	52.88	52.98	53.01	53.04	53.06	53.06
	Aft.	41.83	41.34	43.69	45.01	46.55	47.24	47.81	48.08	48.20	48.25	48.29	48.30	48.31	48.31	48.31
601	Bef.	41.97	41.22	44.15	46.14	48.68	49.97	51.52	52.12	52.48	52.75	52.89	52.90	52.93	52.94	52.95
	Aft.	41.70	41.06	43.47	44.96	46.61	47.30	48.01	48.25	48.39	48.48	48.53	48.54	48.55	48.55	48.55

PNG																
001	Bef.	38.78	38.05	42.94	43.94	48.10	50.04	53.84	56.05	59.64	62.35	66.37	68.80	72.36	74.01	80.24
	Aft.	28.95	28.96	28.92	28.92	28.91	28.91	28.91	28.90	28.90	28.90	28.90	28.90	28.90	28.90	28.90
003	Bef.	38.02	37.39	41.30	43.22	47.20	49.35	52.95	55.29	58.69	61.17	65.52	68.35	70.81	73.75	78.57
	Aft.	28.99	29.00	28.97	28.96	28.95	28.95	28.95	28.95	28.95	28.95	28.95	28.95	28.95	28.95	28.95
050	Bef.	37.34	36.81	41.30	42.74	46.43	48.89	52.32	54.77	58.10	60.86	64.01	67.77	70.21	73.00	79.44
	Aft.	29.00	29.00	28.97	28.97	28.96	28.96	28.95	28.95	28.95	28.95	28.95	28.95	28.95	28.95	28.95
601	Bef.	37.15	36.55	40.31	42.64	46.30	48.58	52.12	54.49	57.70	60.66	64.11	67.71	70.96	74.78	76.58
	Aft.	29.02	29.03	28.99	28.99	28.98	28.98	28.98	28.97	28.97	28.97	28.97	28.97	28.97	28.97	28.97

**Table 13:** Bit error rates result on Gaussian noise attack.

#	1	2	3	4	5	6	7	8	9	10	11	12	13	14	15
001	96	96.5	100	100	100	100	100	100	100	100	100	100	100	100	100
003	83	95.6	98.3	99.8	99.8	100	99.8	100	100	100	100	100	100	100	100
050	83	94.3	95.6	97	99.5	100	100	100	100	100	100	100	100	100	100
601	91	94.3	97.5	94.3	100	100	100	100	100	100	100	100	100	100	100
001	100	100	100	100	100	100	100	100	100	100	100	100	100	100	100
003	100	100	100	100	100	100	100	100	100	100	100	100	100	100	100
050	100	100	100	100	100	100	100	100	100	100	100	100	100	100	100
601	100	100	100	100	100	100	100	100	100	100	100	100	100	100	100

**Table 14:** PSNR results on median filtering attack.

#		1	2	3	4	5	6	7	8	9	10	11	12	13	14	15
JPEG																
001	Bef.	40.74	40.12	43.06	45.01	47.61	48.99	50.65	51.20	51.80	52.05	52.19	52.25	52.27	52.28	52.30
	Aft.	41.81	41.75	42.11	42.20	42.28	42.29	42.31	42.32	42.32	42.32	42.32	42.32	42.33	42.33	42.33
003	Bef.	42.50	41.93	44.86	46.75	49.27	50.69	51.84	52.69	53.00	52.99	53.24	53.27	53.27	53.29	53.28
	Aft.	41.51	41.44	41.87	42.00	42.10	42.13	42.15	42.16	42.16	42.16	42.16	42.16	42.16	42.16	42.16
050	Bef.	42.20	41.61	44.55	46.37	48.90	50.26	51.62	52.35	52.71	52.88	52.98	53.01	53.04	53.06	53.06
	Aft.	41.23	41.17	41.57	41.69	41.78	41.80	41.83	41.84	41.84	41.84	41.84	41.84	41.84	41.84	41.84
601	Bef.	41.97	41.22	44.15	46.14	48.68	49.97	51.52	52.12	52.48	52.75	52.89	52.90	52.93	52.94	52.95
	Aft.	40.93	40.83	41.22	41.32	41.39	41.41	41.44	41.44	41.44	41.45	41.45	41.45	41.45	41.45	41.45

PNG																
001	Bef.	38.78	38.05	42.94	43.94	48.10	50.04	53.84	56.05	59.64	62.35	66.37	68.80	72.36	74.01	80.24
	Aft.	38.66	37.95	42.64	43.57	47.19	48.69	51.13	52.15	53.27	53.78	54.15	54.27	54.35	54.37	54.41
003	Bef.	38.02	37.39	41.30	43.22	47.20	49.35	52.95	55.29	58.69	61.17	65.52	68.35	70.81	73.75	78.57
	Aft.	37.83	37.23	40.90	42.60	45.79	47.22	49.07	49.88	50.62	50.91	51.18	51.26	51.29	51.32	51.34
050	Bef.	37.34	36.81	41.30	42.74	46.43	48.89	52.32	54.77	58.10	60.86	64.01	67.77	70.21	73.00	79.44
	Aft.	37.09	36.58	40.71	41.92	44.72	46.21	47.73	48.45	49.01	49.28	49.44	49.53	49.56	49.57	49.59
601	Bef.	37.15	36.55	40.31	42.64	46.30	48.58	52.12	54.49	57.70	60.66	64.11	67.71	70.96	74.78	76.58
	Aft.	36.84	36.25	39.69	41.65	44.28	45.56	46.99	47.61	48.09	48.33	48.47	48.54	48.57	48.58	48.59

**Table 15:** Bit error rates result on median filtering attack.

#	1	2	3	4	5	6	7	8	9	10	11	12	13	14	15
001	79	87.4	92.2	95.3	98	99.2	99.7	99.7	98.9	100	100	100	100	100	100
003	80	86.4	86.5	92.7	97.7	98.7	99.7	99.7	100	100	100	100	100	100	100
050	79	82.1	93.8	95.1	98.5	98.9	99.7	99.7	100	100	100	100	100	100	100
601	79	87.4	92.5	96.1	98.3	99.1	99.2	100	99.5	100	100	100	100	100	100
001	97	98.6	71.5	99.6	99.7	99.8	99.9	100	100	100	100	100	100	100	100
003	95	96.4	85.1	98.6	99.3	99.4	99.6	100	100	100	100	100	100	100	100
050	94	96.3	73.5	98.5	99.2	99.7	98.1	99.8	99.8	100	100	100	100	100	100
601	93	95.5	93.9	98.6	98.9	99.6	99.7	99.8	99.8	100	100	100	100	100	100
